# Predictors of normalized HbA1c after gastric bypass surgery in subjects with abnormal glucose levels, a 2-year follow-up study

**DOI:** 10.1038/s41598-020-72141-0

**Published:** 2020-09-15

**Authors:** Neda Rajamand Ekberg, Henrik Falhammar, Erik Näslund, Kerstin Brismar

**Affiliations:** 1 Centrum of Diabetes, Stockholm, Sweden; 2 Department of Molecular Medicine and Surgery, D02:04, Karolinska Institutet, 171 76 Stockholm, Sweden; 3 Department of Endocrinology, Metabolism and Diabetes, Karolinska University Hospital, Stockholm, Sweden; 4 Department of Clinical Sciences, Danderyd Hospital, Karolinska Institutet, Stockholm, Sweden

**Keywords:** Endocrinology, Endocrine system and metabolic diseases, Medical research, Outcomes research

## Abstract

Clinical biomarkers can predict normalization of HbA1c after Roux-en-Y gastric bypass (RYGB) surgery, but it is unclear which are the most predictive.The aim of this study was to compare biomarkers for insulin sensitivity and other clinical parameters in the prediction of normalization of HbA1c after RYGB surgery. This study included 99 (23 men) obese subjects (BMI > 35 kg/m^2^) undergoing a laparoscopic RYGB. Clinical and biochemical examinations were performed pre-operatively and up to 2 years after surgery. Pre-operatively, normal fasting glucose levels were found in 25 individuals (NG), prediabetes in 46 and type 2 diabetes (T2DM) in 28. At baseline IGF-I (SD), IGFBP-1 and adiponectin levels were low while leptin was high. Weight loss was observed in all three groups, most in the prediabetes group. After 2 years HbA1c was decreased in prediabetes and T2DM. In all three groups insulin, HOMA-IR, lipids and blood pressure improved, IGFBP-1 and adiponectin increased and leptin decreased. IGF-I (SD) increased only in T2DM. In those with prediabetes or T2DM (n = 74), HbA1c at 2 years correlated to baseline BMI (r = -0.27, p = 0.028), age (r = 0.43, p < 0.001), HbA1c (r = 0.37, p = 0.001) and IGFBP-1 (r = 0.25, p = 0.038), and was normalized in 45/74 (61%) at 1 year and in 36 subjects (49%) at 2 years. These responders were younger, had higher BMI, larger waist circumference, lower HbA1c and lower IGFBP-1 levels at baseline. In a multiple regression model age (negative, p = 0.021) and waist circumference (positive, p = 0.047) were the only predictors for normalized HbA1c. RYGB normalized HbA1c in 49% at two years follow-up, which was predicted by low baseline IGFBP-1 level, a marker of hepatic insulin sensitivty and insulin secretion. However,. younger age and larger waist circumference were the only predictors of normalized HbA1c in multivariate analysis.

## Introduction

The incidence of obesity is increasing at an alarming rate worldwide^1^. Obesity is associated with premature mortality and risk of comorbidities such as insulin resistance (IR), type 2 diabetes mellitus (T2DM), hypertension, cardiovascular disease, musculoskeletal disorders and some types of cancer^2^. Lifestyle modification and pharmacological therapy result in insufficient long-term weight loss in most obese patients, leaving bariatric surgery as a possible method to achieve long-term substantial weight loss^3–5^. There are several, randomized studies which clearly demonstrate that bariatric surgery is superior to best medical practice in treatment of severe obesity related comorbidities. In addition, long-term, non-randomized studies have demonstrated significant benefits of bariatric surgery on comorbidities^6,7^. One main purpose of bariatric surgery is to normalize blood glucose and/or reduce IR and thus prevent future cardiovascular disease and T2DM^8^. Since there are some severe side effects as well as recurrence of T2DM after bariatric surgery^9,10^, it is important to find simple predictors to identify which subjects will benefit the most from bariatric surgery with normalization of HbA1c and/or insulin sensitivity. It has been shown that fasting insulin level can be a better predictor than BMI of the outcome^11^.

Serum biomarkers of insulin sensitivity and insulin secretionare are adiponectin, insulin-like growth factor (IGF) bindingprotein-1 (IGFBP-1), IGF-I and leptin^12–19^. Insulin regulates the synthesis of IGFBP-1, which is one of the binding proteins for IGF-I regulating its activity^14^. IGF-I, regulated by growth hormone, insulin and amino acid, is of importance for glucose homeostasis through enhancing glucose uptake in muscles and by increasing insulin sensitivity^14^. IGFBP-1, produced in the liver, has both IGF dependent and independent effects^20^, which may explain the finding that conditions characterized by hyperinsulinemia such as insulin resistance and metabolic syndrome are associated with decreased levels of IGFBP‐1^18,21,22^. Fasting IGFBP-1 is a marker of hepatic insulin sensitivity^23^ and low levels of IGFBP-1 predict development of prediabetes and T2DM^24–27^. In T2DM the IGFBP-1 levels are increased due to beta-cell dysfunction with decreased portal insulin delivery to the liver. Adiponectin produced in the adipose tissue negatively regulated by inflammatory cytokines is also marker of insulin sensitivity and low levels predict T2DM^12,16^. Leptin produced in the adipose tissue and a marker of adiposity and insulin sensitivity^4,15^. High levels are associated with both leptin resistance and Insulin resistance^4^.

The aim of this study was to evaluate biomarkers of insulin sensitivity and other clinical parameters which can predict normalization of HbA1c as primary enpoint and improvement of insulin sensitivity as secondary endpoint after Roux-en-Y gastric bypass (RYGB) surgery in subjects with normal glucose levels (NG), prediabetes (preDM) or T2DM. Thus, we have studied fasting levels of IGF-I, IGFBP-1, adiponectin and leptin as well as HOMA-IR before and 1 year after surgery and anthropometric data and metabolic factors before, 1 and 2 years after surgery.

## Results

### Baseline characteristics

There were no differences between the three groups with regard to BMI, weight or waist circumference (Table 1). The T2DM and PreDM groups were significantly older (p < 0.001 for both groups), and had significantly higher fasting plasma (fP) glucose (p < 0.001 for both groups), HbA1c (p < 0.001 for both groups), fasting serum (fS) insulin and HOMA-IR compared to the NG group. T2DM had significantly higher glucose (p < 0.001) and HbA1c (p < 0.001) levels compared to the PreDM group.

**Table 1:** Clinical characteristic of the subjects at baseline before Roux-en-Y gastric bypass. Data is shown as mean ± SEM or median (IQR).

Groups according glucose tolerance	NG n = 25 (25%)	PreDM n = 46 (46%)	T2DM n = 28 (28%)	P-values
Women	n = 22 (88%)	n = 32 (70%)	n = 22 (79%)	
Men	n = 3 (12%)	n = 14 (30%)	n = 6 (21%)	
Age (year)	38.2 ± 1.7	48.4 ± 1.6	52.8 ± 1.6	0.001
Weight (kg)	113.6 ± 3.0	123.0 ± 4.1	118.0 ± 3.8	ns
Waist (cm)	118.9 ± 2.4	129.0 ± 2.7	128.7 ± 1.9	ns
BMI (kg/m^2^)	41.4 ± 0.9	43.0 ± 1.2	41.2 ± 0.8	ns
fP-glucose (mmol/L)	5.2 ± 0.04	5.8 ± 0.1	8.6 ± 0.4	< 0.001
HbA1c (mmol/mol)	35 ± 0.4	40 ± 0.6	57 ± 3.2	< 0.001
fS-insulin (mU/L)	6.7 (5.4–11.5)	11.2 (8.1–16.4)	13.4 (7.7–19.1)	0.048
HOMA-IR	2.2 ± 0.4	3.2 ± 0.3	5.3 ± 0.5	< 0.001
	n = 14	n = 27	n = 20	

*NG* normal glucose levels, *PreDM* prediabetes, *T2DM* type 2 diabetes, *ns* non-significant, *BMI* body mass index, *HOMA-IR* homeostatic model assessment of insulin resistance, *f* fasting, *P* plasma, *S* serum.

### Baseline levels of biomarkers of insulin sensitivity after 2 weeks of caloric restriction

There were no differences between the three groups with regards to mean IGF-I (SD), IGFBP-1, leptin or adiponectin at baseline (Table 2). Therefore, we analysed the mean levels of biomarkers at baseline for all 99 subjects together. In the whole group the mean IGF-I (SD) level, IGFBP-1 and adiponectin level were all low while the mean leptin level was high (Table 2). Four (4%) of the subjects had normal leptin levels (BMI 35.6 ± 2.6 kg/m^2^), 21 (21%) had normal adiponectin (BMI 41.4 ± 1.5 kg/m^2^) and 33 (33%) had normal IGFBP-1 levels after 2 weeks of caloric restriction.

**Table 2:** Markers of insulin secretion and insulin sensitivity at baseline preoperatively, mean ± SEM.

	NG (n = 25)	PreDM (n = 46)	T2DM (n = 28)	P-value	All (n = 99)	Normal levels (middle aged healthy people)
IGF-I pre-operative (SD)	− 1.0 ± 0.3	− 0.8 ± 0.2	− 1.3 ± 0.3	ns	− 1.0 ± 0.1	± 2
IGFBP-1 pre-op (µg/L)	17.8 ± 2.1	18.2 ± 2.0	21.1 ± 2.7	ns	18.9 ± 1.3	40–46 (females)
						27–31 (males)
Leptin pre-op (µg/L)	37.6 ± 3.3	39.0 ± 3.5	35.3 ± 3.0	ns	37.6 ± 2.0	13–20 (females)
						7–9 (males)
Adiponectin pre-op (mg/L)	7.9 ± 0.5	7.6 ± 0.6	6.5 ± 0.6	0.062	7.4 ± 0.4	13–15 (females)
						8–9 (males)

*NG* normal glucose levels, *PreDM* prediabetes, *T2DM* type 2 diabetes, *ns* not significant.

### Metabolic improvements 1 and 2 years after RYGB

Significant weight loss and decrease in BMI were observed in all three groups at 12 and 24 months after RYGB (Table 3), which was accompanied by significant decrease in waist circumference and improvement in fP-glucose and HbA1c at 12 months. The PreDM group had lost most weight compared to the NG and T2DM groups. After 24 months the mean HbA1c level in subject with normal glucose levels (NG) was at the baseline level, while the mean HbA1c levels in subject with PreDM and T2DM were lower compared to the pre-operative levels (p < 0.001 for both groups)(Table 3). Mean plasma levels of insulin were decreased 12 months after RYGB in all three groups. Mean values of insulin sensitivity determined as HOMA-IR improvd to normal values in all three groups. The lipid profiles were improved in all three groups at 12 months after surgery and remained improved after 24 months except for LDL in T2DM. The systolic and diastolic blood pressure was significantly lower at 12 and 24 months (Table 3).

**Table 3:** Clinical findings 1 and 2 years after Roux-en-Y gastric bypass in subjects with normal glucose, prediabetes and type 2 diabetes at baseline. Data are presented as mean ± SEM.

	Pre-operation	1 year post RYGB	2 years post RYGB	P-value
**Weight (kg)**				
NG	113.6 ± 3.0	74.6 ± 2.3	76.8 ± 2.6	< 0.001
PreDM	123.0 ± 4.1	82.7 ± 2.6	80.2 ± 2.7	< 0.001
T2DM	118.0 ± 3.8	82.2 ± 2.4	83.6 ± 3.0	< 0.001
**Waist (cm)**				
NG	118.9 ± 2.4	82.6 ± 18	NA	< 0.001
PreDM	129.0 ± 2.7	92.1 ± 2.1	NA	< 0.001
T2DM	128.7 ± 1.9	94.0 ± 2.1	NA	< 0.001
**BMI (kg/m**^**2**^**)**				
NG	41.4 ± 0.9	26.5 ± 0.8	27.4 ± 0.9	< 0.001
PreDM	43.0 ± 1.2	28.7 ± 0.7	28.1 ± 0.7	< 0.001
T2DM	41.2 ± 0.8	28.8 ± 0.7	29.2 ± 0.9	< 0.001
**fP-Glucose (mmol/L)**				
NG	5.2 ± 0.04	4.8 ± 0.1	4.7 ± 0.1	< 0.001
PreDM	5.8 ± 0.1	5.0 ± 0.1	5.0 ± 0.1	< 0.001
T2DM	8.6 ± 0.4	5.5 ± 0.2	5.4 ± 0.2	< 0.001
**HbA1C (mmol/mol)**				
NG	35 ± 0.4	34.4 ± 0.7	35.3 ± 0.8	0.701
PreDM	40 ± 0.6	36 ± 0.5	37 ± 0.5	< 0.001
T2DM	57 ± 3.2	39 ± 0.9	40 ± 1.0	< 0.001
**Fasting P-Insulin (mU/L)**				
NG (n = 15)	9.2 ± 1.5	4.0 ± 0.4	NA	< 0.001
PreDM (n = 27)	12.0 ± 1.2	5.2 ± 0.7	NA	< 0.001
T2DM (n = 20)	13.7 ± 1.3	5.3 ± 0.7	NA	< 0.001
**HOMA-IR**				
NG (n = 14)	2.2 ± 0.4	0.9 ± 0.09	NA	< 0.001
PreDM (n = 27)	3.2 ± 0.3	1.1 ± 0.2	NA	< 0.001
T2DM (n = 20)	5.3 ± 0.5	1.3 ± 0.2	NA	< 0.001
**Triglycerides (mmol/L)**				
NG	1.5 ± 0.3	0.9 ± 0.1	0.9 ± 0.1	< 0.001
PreDM	1.6 ± 0.1	1.0 ± 0.1	0.9 ± 0.1	< 0.001
T2DM	1.9 ± 0.1	1.2 ± 0.1	1.3 ± 0.1	< 0.001
**LDL cholesterol (mmol/L)**				
NG	3.2 ± 0.1	2.3 ± 0.1	2.5 ± 0.2	< 0.001
PreDM	3.3 ± 0.2	2.4 ± 0.1	2.6 ± 0.1	< 0.001
T2DM	2.9 ± 0.2	2.5 ± 0.1	2.8 ± 0.1	0.089
**HDL cholesterol (mmol/L)**				
NG	1.2 ± 0.1	1.6 ± 0.1	1.7 ± 0.1	< 0.001
PreDM	1.1 ± 0.1	1.6 ± 0.1	1.6 ± 0.1	< 0.001
T2DM	0.9 ± 0.1	1.4 ± 0.1	1.4 ± 0.1	< 0.001
**Systolic BP (mmHg)**				
NG	138.5 ± 2.5	121.2 ± 2.1	118.6 ± 2.2	< 0.001
PreDM	141.8 ± 2.5	126.1 ± 2.2	128.2 ± 2.8	< 0.001
T2DM	150.9 ± 3.8	131.4 ± 3.0	135.5 ± 3.4	0.044
**Diastolic BP (mmHg)**				
NG	83.3 ± 1.3	78.6 ± 1.1	79.7 ± 1.7	< 0.001
PreDM	83.3 ± 1.3	78.6 ± 1.1	79.7 ± 1.7	0.049
T2DM	85.9 ± 1.8	79.3 ± 1.4	81.3 ± 1.7	0.026

*NG* normal glucose levels, *PreDM* prediabetes, *T2DM* type 2 diabetes, *RYGB* Roux-en-Y gastric bypass, *NA* not available, *HOMA-IR* homeostatic model assessment of insulin resistance, *BP* blood pressure.

### Changes in biomarkers of insulin secretion and sensitivity (IGF-I, IGFBP-1, adiponectin and leptin) 1 year after RYGB surgery

IGF-I (SD) increased significantly after RYGB in T2DM but not in the NG and PreDM groups. Mean IGFBP-1 and adiponectin levels increased significantly to normal values in all three groups (p < 0.001 for all groups) and leptin decreased significantly in all three groups (p < 0.001 for all groups)(T able 4).

**Table 4:** Changes in biomarkers of insulin secretion and insulin sensitivity postoperatively, mean ± SEM.

	NG (n = 25)	PreDM (n = 46)	T2DM (n = 28)	P-value
**IGF-I (SD) pre-operative**	− 1.0 ± 0.3	− 0.8 ± 0.2	− 1.3 ± 0.3	Ns
**IGF-I (SD) 1-year post-op**	− 0.7 ± 0.2	− 1.0 ± 0.2	− 0.5 ± 0.3	Ns
Differences compared to pre-op	ns	ns	p = 0.015	
**IGFBP-1 pre-op (µg/L)**	17.8 ± 2.1	18.2 ± 2.0	21.1 ± 2.7	ns
**IGFBP-1 1-year post-op (ug/L)**	49.0 ± 4.5	49.0 ± 4.2	51.4 ± 5.3	ns
Differences compared to pre-op	P < 0.001	P < 0.001	P < 0.001	
**Leptin pre-operative (µg/L)**	37.6 ± 3.3	39.0 ± 3.5	35.3 ± 3.0	ns
**Leptin 1-year post-op (µg/L)**	12.0 ± 2.3	11.7 ± 1.5	13.8 ± 2.2	ns
Differences compared to pre-op	P < 0.001	P < 0.001	P < 0.001	
**Adiponectin pre-operative (mg/L)**	7.9 ± 0.5 (8.3, 3.7–13.3)	7.6 ± 0.6 (6.4, 1.4–19.1)	6.5 ± 0.6 (5.5, 2.3–15.9)	P = 0.062
**Adiponectin 1-year post-op (mg/L)**	15.9 ± 1.1	13.9 ± 1.0	12.4 ± 1.0	ns
Differences compared to pre-op	P < 0.001	P < 0.001	P < 0.001	

*NG* normal glucose levels, *PreDM* prediabete, *T2DM* type 2 diabetes, *ns* not significant.

### Correlations between clincal parameters including markers of insulin sensitivity/secretion at baseline-and HbA1c 2 years after RYGB

In those with abnormal glucose levels, the combined group of PreDM and T2DM (n = 74), the HbA1c levels 2 years after surgery correlated inversely to baseline BMI (r = − 0.27, p = 0.028) and positively to preoperative age (r = 0.43, p < 0.001), preoperative HbA1c (r = 0.37, p = 0.001) and to IGFBP-1 (r = 0.25, p = 0.038) but not to preoperative glucose, IGF-I (SD), lipids, adiponectin, insulin levels or HOMA-IR. There was a tendency to an inverse correlation between preoperative leptin levels and HbA1c 2 years after surgery (r = − 0.23, p = 0.060).

### Comparisons between those with and without normalized HbA1c at 1 and 2 years

The HbA1c levels were normalized (< 39 mmol/mol) in 45 out of 74 (61%) subjects with abnormal glucose levels 1-year post-op. The number decreased to 36 subjects (49%) 2 years post-op. The group with normalized HbA1c 2 years after RYGB were at baseline compared to to the group who did not normalize HbA1c significantly younger (mean age 46.8 ± 1.7 years vs 54.0 ± 1.6 years, p = 0.004), had significantly higher BMI (mean 44.0 ± 1.2 kg/m^2^ vs 40.6 ± 5.6 kg/m^2^, p = 0.040), larger waist circumference (mean 133.1 ± 2.7 cm vs 124.5 ± 2.5 cm, p = 0.028), lower HbA1c (mean 44.9 ± 2.1 mmol/mol compared to 49.4 ± 2.7 mmol/mol, p = 0.027) and lower IGFBP-1 levels (mean 18.0 ± 2.3 μg/L compared to 21.6 ± 2.7 μg/L, p = 0.050). All other biomarkers were at baseline not different between responders and non-responders.

In a stepwise multiple regression model with preoperative age, BMI, waist, glucose, HbA1c, HOMA-IR, IGFBP-1, leptin and adiponectin, the only significant predictors were age (unstandardized beta value -0.0672, p = 0.021) and waist (unstandardized beta value 0.0417, p = 0.047) of normalized HbA1c 2 years after surgery. IGFBP-1 levels was at baseline significantly correlated inversely to insulin (r = − 0.45, p < 0.001), BMI (r = − 0.26, p = 0.026), waist circumference (r = − 0.26, p = 0.012), and positively to age (r = 0.404, p < 0.001), adiponectin (r = 0.44, p < 0.001) and HDL-cholesterol (r = 0.36, p < 0.001) but not to glucose, HbA1c, triglycerides, LDL-cholesterol or blood pressure.

Seventeen out of the 25 subjects (68%) with normal glucose control (NG) had low IGFBP-1 before RYGB of whom 15 (92%) had increased their levels above 20 µg/L 1 year after RYGB, thus 2 (8%) subjects continue to have low levels. Eighteen out of 20 subjects (90%) had normalized adiponectin levels.

In the group with normal glucose the subgroup with low IGFBP-1 levels (≤ 20 µg/L) (n = 15) had preoperatively increased HOMA-IR and a tendency to increased weight, insulin levels, triglycerides and diastolic blood pressure compared to the subgroup with high IGFBP-1 (> 20 µg/L). Similarly, in the group with PreDM the subgroup with low IGFBP-1 levels (n = 32) at baseline had signs of the metabolic syndrome with decreased HDL and adiponectin as well as increased triglycerides, insulin, HOMA-IR and IGFSD compared to the subgroup with high IGFBP-1 (n = 13). Moreover, the subgroup with low IGFBP-1 at baseline had after 1 year, in spite of significant weight loss, lower adiponectin and IGFBP-1 levels. Thus, those with low IGFBP-1 at baseline had increased risk for future T2DM. Futhermore, patients with T2DM and low IGFBP1 levels (n = 17) had after 1 and 2 years still signs of the metabolic syndrome in contrast to the patients with T2DM and high IGFBP-1 (n = 11).

At baseline 4 subjects (4%) had low-normal levels of leptin in spite of obesity, which suggest relative leptin deficiency as a cause of obesity. The delta weight change after 1 and 2 years were negatively associated to baseline IGFBP-1 (1 year p = 0.006; 2 year p = 0.064), but not to leptin and adiponectin.

## Discussion

The prevalence of prediabetes (46%) and T2DM (28%) were common in this cohort of obese subjects selected because of high BMI. The fasting glucose levels were high in spite of prior 2 weeks caloric restriction. Other studies of bariatric surgery have reported similar number of obese subjects to have prediabetes or T2DM and that bariatric surgery is particular effective in treating T2DM^11,28,29^.

In this study significant weight loss was observed in obese subject with normal glucose, preDM and T2DM at 12 and 24 months after RYGB. In parallel with weight loss significant improvement in the risk markers measured (fP-glucose, HbA1c, lipids and blood pressure) was observed including all markers of insulin resistance. These results are in line with previous studies^30^. IGFBP-1 was the only biomarker at baseline which predicted normalization of HbA1c. However, in a stepwise multiple regression model younger age and larger waist circumference at baseline were the only predictor for normalization of HbA1c when corrected for confounders. However, since baseline IGFBP-1 correlated to both age and waist circumference before surgery this may explain this finding.

Investigating markers of insulin sensitivity (IGFBP-1, HOMA-IR, leptin and adiponectin) showed that baseline levels of IGFBP-1 and adiponectin were low in the three groups despite prior caloric restriction for 2 week. This suggests presence of hyperinsulinemia and insulin resistance in the majority of subjects. In a previous study strict caloric restriction for a shorter period reduced insulin secretion, and IGF-1 levels and increased IGFBP-1 both in overweight NG subjects and in T2DM^31^. It is known that 2 weeks hypocaloric diet improves hepatic insulin sensitivity greatly which is associated with the increase in IGFBP-1^23^. This indicate that the subjects in the present study probably before the calorie restriction had even lower baseline fasting IGFBP-1 and higher insulin and IGF-I SD levels than the present data. Also adiponectin levels increase with hypocaloric diet^32^, thus our study goups most probably had lower levels before the 2 weeks of hypocaloric diet. Consequently , the majority of the NG and prediabetes subjects in our study had a high risk of future prediabetes or T2DM.

The majority (61%) of the subjects with abnormal glucose levels had normalized HbA1c after 1 year and somewhat less (49%) after two years, although the mean weight/BMI was not changed at 2 years. A sustained improvement of glucose and weight has been shown post-RYGB in patients with and without T2DM^33^. It is belived that both weight loss and improved beta-cell function contribute to the sustained improved glycemic control.

IGFBP-1 was the only biomarker at baseline which predicted normalization of HbA1c. Since IGFBP-1 levles is determined by insulin secretion our finding support previous report on fasting insulin as a better predictor than BMI for the beneficial effect of RYGB on metabolic control^11^. Interestingly, in addition, the lower baseline IGFBP-1, the higher weight loss after RYGB. Low IGFBP-1 is associated with hepatic insulin resistance and hyperinsulinemia. Our study suggests that only those with preoperatively beta-cells with remaining capacity to produce enough insulin when needed may normalize the metabolic control after improvement of hepatic insulin resistance. This is in line with the recent study by Jorgensen et al.^33^. It has been shown that improvement of hepatic insulin resistance is an important factor in combination with adequate postprandial insulin response explaining the metabolic effect of RYGB^34,35^. Weght loss, reduced insulin secretion and improved hepatic insulin sensitivity are associated with inceased IGFBP-1 levels^29,36,37^. Indeed, weight loss was accompanied by increased mean levels of both IGFBP-1 and adiponectin 1 year after the surgery.

In our cohort low adiponectin did not predict normalization of HbA1c after weight loss, which may be due to the fact that adiponectin is a marker of insulin resistance in adipose tissue, muscle and the whole body and not of beta-cell function^38^. In contrats to IGFBP-1, adiponectin nor leptin levels do not indicate beta-cell function per se.

The majority of the 71 subjects with abnormal glucose levels before surgery had low levels of IGFBP-1 (n = 49) and adiponectin (n = 56). It has previously been shown that low fasting IGFBP-1 and adiponectin levels predict future T2DM, especially in those with increased waist circumference^25,26,39^.

IGF/IGF-I (SD) levels were low in all groups before surgery, most probably due both to the caloric restriction and reduced GH secretion in obesity. However, the levels were not significantly changed in spite of weight loss after 1 year except for that in patients with T2DM who showed somewhat increased levels, compared to that before surgery. Thus, IGF-I was not responsible for improvement in HbA1c, which is in line with previous study by Brynskov et al*.*^37^*.*

In multivariate analysis only younger age and larger waist circumference were predictive parameters of HbA1c normalization at 2 years of follow-up. Younger age has been shown to be a predictor of weight loss in bariatric surgery in previous studies^40^. Intuitively, younger age should be a predictor since the glycaemic disturbances should not have been present as long as in elderly and the beta-cells more functional. Thus, HbA1c normalization would be easier with weight loss. Why a high waist circumference will predict a beneficial metabolic effect of RYGB is not fully explained by the present study. Unless the explaination is that the increased waist is a surrogate marker of liver steatosis, hepatic insulin resistance, and low IGFBP-1 levels^23^ which is corrected by RYGB reducing liver steatosis thus improving insulin sensitivity and glucose tolerance. In summary our study suggests that the metabolic control will be improved, even normalized, by RYGB particularly in subjects with low IGFBP-1 levels, which is a biomarker of liver steatosis, hepatic insulin resistance and preserved beta-cell function. However, both age and waist circumference are easier parameters to collect preoperatively compared to analysis of IGFBP-1 serum levels.

At baseline 4% had low-normal levels of leptin in spite of obesity, which suggest relative leptin deficiency as a cause of obesity. The delta weight change after 1 and 2 years were not associated to baseline leptin levels. These subjects may benefit more of treatment with the new synthetic leptin drug^41^.

The increase in mean IGFBP-1 and adiponectin levels 1 year after RYGB to normal levels suggest reduced hepatic inuslin resistance, improved insulin sensitivity and protection against future prediabetes and/or T2DM in those with normal glucose levels and prediabetes^12,42,43^.

There are several clinical scoring systems to predict longterm T2DM remission after RYBG relying on basic clinical parameters such as age, BMI, HbA1c, and the use of insulin therapy and oral hypoglycemic agents, and sometimes C-peptid^44^. How less conventional biomarkers, which we have included in the present study (e.g., IGF-I, IGFBP-1, adiponectin and leptin) would perform if included in such scoring system are unclear but would be worth evaluating in future studies of larger cohorts.

This study had several limitations. Like all retrospective studies the design has its inherent disadvantages, particularly ascertainment bias and negative selection bias. The follow-up time was only 2 years with all hormone levels only available at 1 year. Moreover, the number of included individuals were limited, especially in the three subgroups. Another limitation is that we do not know the levels of the biomarkers as well as the weight and waist circumference before the 2 weeks of calorie restriction. These weeks of moderate calorie restriction should have improved insulin, insulin sensitivity, lipid profile and glucose levels. It would be of interest to see if the response to short-term calorie restriction can predict the response to RYGB. The strength of the study is the availability of many hormones preoperatively and at least at 1 year of follow-up.

In conclusion, this study suggests that the majority of subjects with low fasting IGFBP-1 and adiponectin levels benefit from bariatric surgery with improved insulin sensitivity and glucose tolerance as well as reduced risk factors for cardiovascular disease and future abnormal glucose tolerance. IGFBP-1 was the only biomarker that predicted normalization of HbA1c, supporting that preserved beta-cell function is of importance. However, in multivariate analysis corrected for confounders younger age and larger waist circumference were the only predictors of normalized HbA1c 2 years after RYGB.

## Material and methods

This is a retrospective clinical study of 99 consecutive (23 men) obese subjects referred for laparoscopic RYGB during 2 years. Inclusion criteria was a BMI greater than 35 and all were studied after an overnight fast.

In this cohort 25 subjects (3 men) had normal glucose levels (NG) (fasting plasma-glucose < 5.6 mmol/L and HbA1c < 39 mmol/mol), 46 subjects (14 men) had PreDM (fasting glucose 5.6–6.9 mmol/L and/or HbA1c 39–47 mmol/mol) and 28 subjects (6 men) had T2DM (fasting P-glucose ≥ 7 mmol/l and/or Hba1c ≥ 48)^45^. The 28 subjects with T2DM were treated with diet only (n = 10), metformin (n = 16), sulfonylurea (n = 1), thiazolidinedione (n = 2) and/or insulin (n = 5).

The subjects were studied prior to and 12 and 24 months after RYGB. They were asked to adhere to caloric restriction with 1,000 kcal/day during 2 weeks prior to the surgery, when a standard 100 cm laparoscopic RYGB was performed^46^. Clinical characteristic of the subjects at baseline before RYGB after 2 weeks of recommended caloric restriction are shown in Table 1.

Plasma glucose, serum lipid profile and HbA1c were analysed according to routine methods. HbA1c was determined using the MonoS method, Unimate (Roche Diagnostics, Basel, Switzerland). The values obtained with the MonoS method was recalculated to IFCC standard (mmol/mol): IFCC = (10.11*Mono-S) − 8.94. Normal HbA1c were 27–42 mmol/mol (< 50 years) and 31–46 mmol/mol (≥ 50 years), respectively. Serum insulin levels were determined using the ELISA technique (DakoCytomation). Serum IGF-I was determined by an in-house RIA after separation of IGFs from IGFBPs^47^. The detection level of the RIA was 3.0 µg/L. Cross-reactivity with IGFBP-2 and -3 was < 0.5 and < 0.05%, respectively. To minimize interference of remaining IGFBPs, des(1–3) IGF-I was used as radio-ligand. The intra- and inter-assay CV were 4% and 11%, respectively. Serum levels of IGF-I decrease with age, and are thus expressed as standard deviation (SD) score = [(10logIGF-I-observed + 0.00693 * age) − 2.581]/0.120^48^. Serum IGFBP-1 (μg/L) was analyzed with an in-house RIA^49^. The sensitivity of the RIA was 3 µg/L and the intra- and inter-assay CV were 3% and 10%, respectively. Leptin (μg/L) and adiponectin (mg/L) were analysed with double antibody RIA (Linco Research, St. Charles, MO). Homeostatic Model Assessment of Insulin Resistance (HOMA-IR)*,* a measure of fasting whole body insulin sensitivity, was calculated according to Matthew et al*.*^50^ from fasting serum insulin and blood glucose levels using a computer-solved homeostasis model assessment: HOMA-IR = (fasting insulin x fasting glucose/22.5). Blood pressure was measured after 10 min of rest with patient sitting. Body mass index (BMI) was determined by body-weight (kg)/height (m^2^). Waist circumference was measured standing at the horizontal level two cm above umbilicus.

For middle age healthy NG females normal serum levels were for adiponectin 13–15 mg/L, for leptin 13–20 µg/L, and for IGFBP-1 40–46 µg/L^15,49^. For middle age healthy NG males normal serum levels were for adiponectin 8–9 mg/L, for leptin 7–9 µg/L and for IGFBP-1 27–31 µg/L^15,42^.

All procedures performed in studies involving human participants were in accordance with the ethical standards of the institutional and/or national research committee and with the 1964 Helsinki declaration and its later amendments or comparable ethical standards. The study was approved by the Stockholm Ethical Committee and all subjects gave written informed consent to participate.

### Statistics

Statistical analyses were carried out using STATISTICA software, version 13 (StatSoft, Tulsa, OH, USA). P-values < 0.05 were considered statistically significant. Variables are presented as mean ± SEM. Normality of variables was tested using the Kolmogorov–Smirnov and Lilliefors tests. Differences between 2 dependent values were tested using Wilcoxon Matched Pair test. Differences between 2 groups were tested using Mann-Whiney test. Differences between the three groups’ variables were tested using Kruskal–Wallis ANOVA and Median test. Differences within each group over the 2 years were tested using Friedman ANOVA. Correlations were tested using Spearman Rank Order Correlation. Logistic regressions were preformed to test which parameters affected primary outcome.
